# The Influence of Probiotics on the Firmicutes/Bacteroidetes Ratio in the Treatment of Obesity and Inflammatory Bowel disease

**DOI:** 10.3390/microorganisms8111715

**Published:** 2020-11-01

**Authors:** Spase Stojanov, Aleš Berlec, Borut Štrukelj

**Affiliations:** 1 Faculty of Pharmacy, University of Ljubljana, SI-1000 Ljubljana, Slovenia; spase.stojanov@ijs.si (S.S.); ales.berlec@ijs.si (A.B.); 2 Department of Biotechnology, Jožef Stefan Institute, SI-1000 Ljubljana, Slovenia

**Keywords:** probiotics, Firmicutes, Bacteroidetes, dysbiosis, obesity, inflammation

## Abstract

The two most important bacterial phyla in the gastrointestinal tract, Firmicutes and Bacteroidetes, have gained much attention in recent years. The Firmicutes/Bacteroidetes (F/B) ratio is widely accepted to have an important influence in maintaining normal intestinal homeostasis. Increased or decreased F/B ratio is regarded as dysbiosis, whereby the former is usually observed with obesity, and the latter with inflammatory bowel disease (IBD). Probiotics as live microorganisms can confer health benefits to the host when administered in adequate amounts. There is considerable evidence of their nutritional and immunosuppressive properties including reports that elucidate the association of probiotics with the F/B ratio, obesity, and IBD. Orally administered probiotics can contribute to the restoration of dysbiotic microbiota and to the prevention of obesity or IBD. However, as the effects of different probiotics on the F/B ratio differ, selecting the appropriate species or mixture is crucial. The most commonly tested probiotics for modifying the F/B ratio and treating obesity and IBD are from the genus *Lactobacillus*. In this paper, we review the effects of probiotics on the F/B ratio that lead to weight loss or immunosuppression.

## 1. Introduction

In the human body, trillions of microorganisms live in symbiosis with the host and are mainly located in the gastrointestinal tract, skin, saliva, oral mucosa, conjunctiva, and vagina [1]. Microorganisms that inhabit the gastrointestinal tract (i.e., gut microbiota) number approximately 1 × 10^14^ [2] and play an essential role in intestinal homeostasis, development, and protection against pathogens. Furthermore, their presence in the gut is associated with immunomodulatory and metabolic reactions [3]. Gut microbiota consists of bacteria, yeasts, and viruses. Bacteria in the gut are represented by more than 1000 species that belong to six dominant phyla: Firmicutes, Bacteroidetes, Actinobacteria, Proteobacteria, Fusobacteria, and Verrucomicrobia. Bacteria from the phyla Firmicutes and Bacteroidetes are the most common, representing 90% of the gut microbiota [4]. The gut microbiota of a healthy individual differs in different parts of the gastrointestinal tract and changes with time due to aging (including infant development) and environmental factors such as dietary habits, lifestyle, and antibiotic consumption. Large differences in microbiota composition exist among individuals, with the differences attributed to age, ethnicity, lifestyle, and diet [4,5]. Different microbiota are classified into three distinct enterotypes [6]. Such variations are considered physiological and consistent with healthy microbiota. Nevertheless, changes in microbiota composition are often related to diseases, also termed dysbioses. However, the causality between altered microbiota and various diseases is often unclear. 

To somewhat simplify the complex issue of microbiota composition, the present review only addressed the two major bacterial phyla in the gastrointestinal tract: Firmicutes and Bacteroidetes. The ratio between these two phyla (the Firmicutes/Bacteroidetes (F/B) ratio) has been associated with maintaining homeostasis, and changes in this ratio can lead to various pathologies. For example, increases in the abundance of specific Firmicutes or Bacteroidetes species lead to obesity and bowel inflammation, respectively [7,8]. The phylum Firmicutes includes Gram-positive bacteria with rigid or semi-rigid cell walls that are predominantly from the genera *Bacillus*, *Clostridium*, *Enterococcus*, *Lactobacillus*, and *Ruminicoccus* [4,9], whilst the phylum Bacteroidetes includes approximately 7000 different species of Gram-negative bacteria that are predominantly from the genera *Bacteroides*, *Alistipes*, *Parabacteroides*, and *Prevotella* [10]. Although there is much focus on the F/B ratio, one should bear in mind that this ratio can be affected by an increase in other phyla and that dysbiotic increases in other phyla do not necessarily change the F/B ratio. The most variable phylum was demonstrated to be Proteobacteria, which contributes to dysbiosis [11] and is correlated with a decrease in Firmicutes and general microbial diversity in inflammatory bowel disease (IBD) [12]. 

Balancing the intestinal ecosystem is an important aspect of maintaining normal human body function, and many therapeutic strategies are designed to achieve an appropriate F/B ratio. The administration of probiotics, prebiotics, synbiotics, phage therapy, fecal transplantation, and bacterial consortium transplantation have all been tested as methods to modulate gut microbiota [13]. The present review thus also focuses on the use of probiotics for balancing the F/B ratio and, consequentially, treating obesity and IBD. 

**Figure 1:**
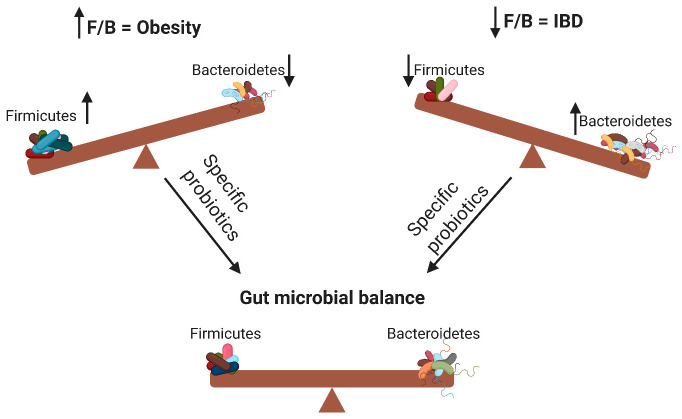
Changes in the Firmicutes/Bacteroidetes (F/B) ratio can cause obesity or inflammatory bowel disease. Specific probiotics can restore the gut microbial balance by influencing the F/B ratio. ↑ means increase; ↓ means decrease.

According to the WHO definition, probiotics are live microorganisms that can confer health benefits to the host when administered in adequate amounts. By modifying the microbiota, probiotics can potentially treat several diseases [14] and exert anti-obesity and anti-inflammatory effects [7]. As illustrated in Figure 1, probiotics contribute to the alteration of the F/B ratio, thereby influencing host obesity or intestinal inflammation. The aim of this review was to elucidate the effects of different probiotic species on obesity and inflammation as well as their relationship with the F/B ratio.

## 2. The Role of Gut Microbiota in Nutrition and Obesity

The gut microbiota plays an essential role in food digestion due to the presence of genes, which encode digestive enzymes that are not present in human cells, but are associated with the metabolism and fermentation of several food compounds that present nutritional benefits to the host. The most abundant metabolic products of gut microbiota are short-chain fatty acids (SCFAs), mainly acetate, propionate, and butyrate, which are produced by anaerobic fermentation of undigested carbohydrates [15]. The gut microbiota also plays a role in metabolizing pharmacologically active compounds such as phytoestrogens [16]. Furthermore, dysbiosis of the gut microbiota can lead to several pathologies including diabetes and obesity [17].

Obesity is a major public health problem that is increasing on a global scale due to the modern lifestyle. It is estimated that by 2030, 38% of the adult population will be overweight, and 20% will be obese [18]. The etiology of obesity is diverse, and many factors play a role in its development. The gut microbiota is involved in the occurrence of obesity by direct interactions with proximal organs or indirect interactions with distant organs through metabolic products (mainly SCFAs) including communication with the liver, adipose tissue, and brain [19]. SCFAs play a crucial role in the development of obesity. They interact with adipose tissue via two G-protein-coupled receptors expressed in adipocytes (Gpr41 and Gpr43); this promotes adipocyte formation and inhibits lipolysis [20]. Furthermore, SCFAs downregulate the synthesis of the hunger-suppressing hormones leptin, peptide YY, and glucagon-like peptide 1 [21]. The gut microbiota can also affect appetite and satiety via vagus nerve activation or immune-neuroendocrine mechanisms [19]. The gut microbiota also promotes bile acid metabolism and modifies hepatic triglyceride and glucose homeostasis through the farnesoid X receptor [22]. Modulating the gut microbiota with different diets and supplementation with probiotics and dietary fibers is a promising approach for the treatment and prevention of obesity [23]. 

### 2.1. The Role of an Increased F/B Ratio in Obesity 

An increased F/B ratio is associated with obesity. Studies have examined the F/B ratio in lean and obese humans and animals using a wide range of biochemical and molecular biological methods in which 16S rRNA gene amplification was the most widely used [24]. One of the first studies that described the correlation between the F/B ratio and obesity was performed in 2005 by analyzing cecal bacteria from homozygous obese (ob/ob), heterozygous obese (ob/+), or homozygous lean (+/+) mice. The F/B ratio was increased in obese mice and decreased in lean mice [25]. Further evidence suggested that colonization of germ-free mice with microbiota from ob/ob or +/+ mice increased their total body fat by 47% or 27%, respectively [26].

An association between the F/B ratio and obesity has also been reported in humans. A recently published systematic review revealed that the majority of studies support a relationship between an increased F/B ratio and obesity [27]. Here, we present some exemplary studies in more detail. A study on stool samples from obese and non-obese Japanese subjects revealed significant differences in their F/B ratios. The percentage of Firmicutes was 37.0 ± 9.1% (in non-obese) and 40.8 ± 15.0% (in obese subjects), whereas the percentage of Bacteroidetes was 44.0 ± 9.8% (in non-obese) and 37.0 ± 14.0% (in obese subjects) [28]. Similar findings were reported in 61 Ukrainian adults in which the F/B ratio was significantly associated with body mass index (BMI). Individuals with a F/B ratio of ≥ 1 were 23% more likely to be overweight than those with a F/B ratio of <1 [29]. Similarly, Qatari subjects (37 obese and 36 lean) exhibited altered gut microbiota; the F/B ratios in obese and lean subjects were 2.25 ± 1.83 and 1.76 ± 0.58, respectively [30]. In Kazakh [31] and Belgian [32] school children, the F/B ratio was significantly higher in the obese groups compared to that of the control groups. In contrast, several studies observed no relationship between the F/B ratio and obesity, weight gain, or BMI. Fecal samples collected from obese (BMI ≥ 30 kg/m^2^) and normal (BMI < 25 kg/m^2^) Korean adolescents (aged 13–16 years) revealed no significant differences in the F/B ratio between the two groups (0.50 ± 0.53 (normal) vs. 0.56 ± 0.86 (obese)). However, there was a difference in the prevalence of two Bacteroidetes genera: *Bacteroides* was 20% more prevalent in the normal group compared to the obese group, and *Prevotella* was 19% more prevalent in the obese group compared to the normal group [33]. Interestingly, in Egyptian subjects, both Firmicutes and Bacteroidetes phyla were increased in the obese group compared to the group with normal weight [34].

To add to the confusion, several studies demonstrated opposite findings (i.e., decreased F/B ratios in obese individuals) [35,36]. There are various reasons for these contradictory results. Presumably, the association between the F/B ratio and obesity varies between specific populations, age groups, genders, and environmental and genetic factors [35], and, as already mentioned, other phyla (such as Proteobacteria) may play an important role. Additionally, there is a limited number of specific bacterial species from the Firmicutes and Bacteroidetes phyla that are associated with obesity. A study on the most important Firmicutes and Bacteroidetes species associated with obesity revealed a strong association between obesity and the following Firmicutes bacteria: *Blautia hydrogenotrophica*, *Coprococcus catus*, *Eubacterium ventriosum*, *Ruminococcus bromii*, and *Ruminococcus obeum*. Conversely, the most common bacteria in lean individuals were *Bacteroides faecichinchillae*, and *Bacteroides thetaiotaomicron* (from Bacteroidetes) and *Blautia wexlerae*, *Clostridium bolteae*, and *Flavonifractor plautii* (from Firmicutes) [36]. These differences were explained by the ability of certain bacteria, especially from the Firmicutes phylum, to produce more enzymes that are responsible for carbohydrate degradation and fermentation. The Firmicutes species *R. bromii* is associated with obesity and utilizes and degrades resistant starch better than *Eubacterium rectale*, *B. thetaiotaomicron*, and *Bifidobacterium adolescentis* [37]. Interestingly, certain Bacteroidetes species also possess a variety of genes for carbohydrate-degrading enzymes [38]. Despite this, the majority of studies support the claim that Firmicutes bacteria have a better capacity to ferment and metabolize carbohydrates and lipids, and thus contribute to the development of obesity. 

### 2.2. Treating Obesity with Probiotics

Manipulating gut microbiota with different dietary supplements can contribute to the restoration of the dysbiotic F/B ratio and treatment or prevention of obesity. Consumed through foods or supplements, probiotics can influence gut microbiota and reduce obesity. However, not all probiotics have the same characteristics as some can even induce weight gain and thus promote obesity. Probiotics that had the potential to reduce the F/B ratio and obesity are mostly bacteria from the genera *Lactobacillus* and *Bacillus* and yeasts from the genus *Saccharomyces* (Table 1).

The administration of *Lactobacillus rhamnosus* GG and *Lactobacillus sakei* NR28 decreased the F/B ratio in obese mice. Furthermore, the two probiotic strains reduced epididymal fat mass, acetyl-CoA carboxylase, fatty acid synthase, and stearoyl-CoA desaturase-1 in the liver [39]. In another study, *L. rhamnosus* GG consumed with a high-fat diet prevented weight gain and decreased the F/B ratio in a C57BL/6J murine model [40]. Treating hyperlipidemic rats with *L. rhamnosus* hsryfm 1301 or its fermented milk for 56 days reduced serum lipid levels, which was associated with recovering gut dysbiosis, and increased Bacteroidetes and decreased Firmicutes abundance by approximately 5% [41]. A 12-week high-fat diet induced an obese insulin-resistant condition in Wistar rats as it increased their body weight, food intake, plasma total cholesterol, and F/B ratio, which induced gut dysbiosis. Oral administration of the probiotic *Lactobacillus paracasei* HII01 together with xylooligosaccharide for 12 weeks reversed the high-fat diet-induced effects by enhancing insulin sensitivity, decreasing low-density lipoprotein cholesterol levels, reducing body weight, and reducing the F/B ratio, thus reversing dysbiosis [42]. 

Probiotic bacteria are well known for their anti-microbial effects against various pathogens. Their anti-microbial mechanisms are diverse; however, producing antibacterial peptides bacteriocins is among the most common. In one study on diet-induced obese mice, bacteriocin-producing *Lactobacillus salivarius* UCC118 Bac+ altered gut microbiota and reduced weight gain more effectively than the non-bacteriocin-producing strain *L. salivarius* UCC118 Bac-. The authors reported increased proportions of Bacteroidetes and Proteobacteria and decreased proportions of Actinobacteria, while the proportion of Firmicutes remained the same [43]. The beneficial effects of *L. salivarius* were also demonstrated in a human clinical trial. *L. salivarius* Ls-33 increased the Bacteroidetes–Prevotella–Porphyromonas–Firmicutes ratio, but did not show any beneficial effects on body weight in obese adolescents [44]. Apart from *Lactobacillus*, bacteria from the genus *Bacillus*, namely *Bacillus amyloliquefaciens* SC06, also decreased the F/B ratio, body weight, and hepatic steatosis in mice consuming a high-fat diet [45].

Although the majority of probiotics are bacteria, other microorganisms are also considered as probiotics. The yeast *Saccharomyces boulardii* has demonstrated positive effects regarding obesity and the F/B ratio. Oral administration of *S. boulardii* to leptin-resistant obese and type 2 diabetic mice for four weeks increased Bacterioidetes by 37%, decreased Firmicutes by 30%, and reduced body weight, fat mass, hepatic steatosis, and inflammatory tone [46]. Similarly, Lei et al. confirmed the relationship between *S. boulardii* and the F/B ratio in mice with D-galactosamine-induced liver injury. Mice treated with *S. boulardii*, in comparison to the control, had a significant increase in Bacterioidetes (61.7% vs. 40.8%) and a decrease in Firmicutes (33.9% vs. 53.7%) [47].

In some cases, the anti-obesity effects of probiotics are not related to the F/B ratio. For example, *Lactobacillus curvatus* HY7601 and *Lactobacillus plantarum* KY 1032 exerted a 38% lower body weight gain, but did not affect the F/B ratio in diet-induced obese mice [48]. In a human double-blind, randomized, placebo-controlled intervention trial, *Lactobacillus gasseri* SBT2055 was administered to subjects with a high BMI. The subjects received fermented milk with or without probiotic, and the body weight (1.4%), BMI (1.5%), waist (1.8%), and hip (1.5%) circumference were significantly decreased in the probiotic group, but not in the control group. Furthermore, the F/B ratio was unaffected [49].

Overall, probiotics cannot be used as a universal therapy against obesity. As previously mentioned, not all probiotics demonstrated the ability to lower the body weight or exert anti-obesity effects. In contrast, *Lactobacillus reuteri* [50] and some bifidobacteria [51] were associated with increased body weight and obesity. For studies of probiotics and their role in obesity, regardless of their effects on the F/B ratio, the reader is referred elsewhere [52,53,54].

## 3. The Role of Gut Microbiota in IBD

IBD represents a group of intestinal disorders, characterized by a complex of inflammatory reactions in the small and large intestine. IBD encompasses two types of inflammatory diseases: ulcerative colitis (UC) and Crohn’s disease (CD). The two diseases are differentiated by their location: UC mainly causes long-lasting inflammation in the colon and rectum, while CD causes inflammation anywhere along the digestive tract, but mostly in the ileum, colon, or both [55]. The exact etiology of these immunological disorders is unclear; however, the gut microbiota is known to importantly contribute to the initiation and progression of IBD. Certain gut bacterial species can adhere to the gut mucosa and invade mucosal epithelial cells, which results in an inflammatory response mediated by the production of tumor necrosis factor-α (TNF-α) by monocytes and macrophages. TNF-α plays a pivotal role in a variety of immunomodulatory reactions, and is associated with IBD via induction of cell apoptosis and necroptosis [56]. The inhibition of TNF-α can improve the health of patients and results in remission of IBD [57,58].

Gut microbiota can also induce inflammatory reactions through Toll-like receptors (TLRs), which play a vital role in gut immunity and are related to the development and progression of IBD. TLRs maintain gut homoeostasis, control immune responses, and shape the microbiota [59]. They are expressed mainly by immune cells; however, several TLRs are also expressed by intestinal epithelial cells and stromal tissue cells of the gastrointestinal tract (TLR-1–9 are expressed in the small and large intestine in humans and mice) [60]. The majority of TLR activators are pathogen-associated molecular patterns (i.e., microbial compounds mainly found in Gram-negative bacteria). The TLR heterodimers TLR-1/2 and TLR-2/6 recognize triacylated and diacylated lipopeptides, respectively [61], while TLR-4 and TLR-5 can be stimulated by lipopolysaccharides and flagellin, respectively [62]. In many cases of IBD, altered TLR expression and TLR mutations were observed, and their inhibition played a beneficial role in slowing down IBD progression [63]. Conversely, certain TLRs play a protective role and can prevent inflammation in the small and large intestine. For example, TLR-9 recognizes bacterial CpG DNA, and its activation correlates with significant improvement in intestinal wound repair and intestinal protection [64].

Additionally, several gut bacteria directly or indirectly influence innate and adaptive immunity and thus evoke or prevent IBD. For example, macrophages in the gut sense microbial signals that trigger them to produce interleukin (IL)-1β [65]. A group of 17 human and 46 murine *Clostridium* species affected regulatory T cell differentiation, function, and accumulation in mice colon, resulting in an anti-inflammatory and protective effect [66]. Gut microbiota metabolites can also protect the host from inflammation. SCFAs increase extrathymic regulatory T cell differentiation through the intronic enhancer CNS1 [67]. Moreover, SCFAs promote the synthesis of the immunosuppressive cytokine IL-10 in T effector cells [68] and combat oxidative stress in the gut [69]. These findings emphasize the important role of SCFAs in gut homeostasis and the fight against IBD. 

### 3.1. The Role of a Decreased F/B Ratio in IBD

Apart from being important factors for obesity, altered F/B ratios were also found in many cases of IBD. In contrast to obesity where increased F/B ratios were observed, decreased F/B ratios have been observed in IBD. One study examined bacterial diversity in fecal samples and identified 13 distinct Firmicutes ribotypes in CD patients (n = 6) and 43 distinct Firmicutes ribotypes in heathy individuals (n = 6) [70]. Similarly, a different study examined the microbial diversity of biopsies from CD and UC patients and healthy controls. A decrease in Firmicutes abundance was observed; more precisely, bacteria from the class Clostridia were decreased in CD patients, but not in UC patients or healthy individuals. Conversely, Bacteroidetes abundance was significantly increased in CD patients compared to UC patients and healthy individuals (74.97%, 64.31%, and 67.41% respectively) [71]. Alterations in the gut microbiota were also associated with disease activity and severity of CD and UC. For example, Firmicutes was less abundant in UC patients with the active disease compared to patients with the inactive disease. Likewise, Firmicutes abundance was significantly decreased in CD patients with the aggressive disease compared to CD patients with the nonaggressive disease [72]. *Faecalibacterium prausnitzii* is a Firmicutes species that was decreased in IBD patients, and its reduction was associated with postoperative recurrence of CD at six months [73]. Furthermore, *F. prausnitzii* demonstrated anti-inflammatory effects in vivo and in vitro. In mice with induced colitis, live *F. prausnitzii* (or their supernatant) reduced the severity of colitis and corrected dysbiosis. In addition, in vitro stimulation of blood mononuclear cells by *F. prausnitzii* caused higher secretion of IL-10 and lower secretion of IL-12 and interferon-γ (IFN-γ) [73].

In contrast, Bacteroidetes bacteria are often increased in IBD and associated with its progression and development. Mucosal biopsies from inflamed and non-inflamed regions of intestine from IBD patients and healthy individuals revealed reduced Firmicutes and increased Bacteroidetes abundance in IBD samples, while Enterobacteriaceae was increased only in CD patients. The biopsies also revealed higher bacterial dysbiosis in inflamed regions compared to non-inflamed regions [74]. An altered gut microbiota and F/B ratio was demonstrated by another study that performed mucosal biopsies and RNA extraction from CD and UC patients. The F/B ratio in both CD and UC patients was significantly decreased compared to that of the control group; additionally, the composition of species, genera, and families was changed. The abundance of *Bacteroides* and *Lactobacillus* was higher in UC patients compared to CD patients and the control group; the abundance of *Escherichia coli* was also increased in UC patients compared to the control group. Conversely, *Clostridium coccoides* and *Clostridium leptum* were reduced in CD patients [75]. Aside from biopsies, gut dysbiosis has also been examined in fecal samples. Fecal microbial samples from Japanese CD patients had a significantly increased abundance of *Bacteroides* compared to that in healthy individuals. Additionally, the abundance of *Faecalibacterium* and *Bifidobacterium* was significantly decreased in CD patients [76]. Similarly, Japanese UC patients hosted bacteria that were not detected in healthy individuals including *Ruminococcus*, *Eubacterium*, *Fusobacterium*, *Gammaproteobacteria*, unclassified *Bacteroides*, and unclassified *Lactobacillus* [77]. Different Bacteroidetes bacteria were associated with varying degrees of IBD. In an antibiotic-pretreated mouse line with defects in transforming growth factor (TGF)βRII and IL-10R2 signaling, severe ulcerative disease was observed following gavage with *Bacteroides vulgatus* and *B. thetaiotaomicron*, while *Bacteroides* sp. TP5 induced only a milder disease consisting of lymphocytic infiltration into the mucosa [78].

Analogous to studies that showed little effect of the F/B ratio on obesity, a decreased F/B ratio was also not observed in all IBD cases. A fecal microbial analysis of IBD patients from Kazan revealed decreased abundancies of Bacteroidetes, Firmicutes, and Verrucomicrobia, while the abundancies of Proteobacteria, Actinobacteria, and Fusobacteria were increased [79]. In 48 UC patients, the *Bacteroides* bacterial copy number in fecal samples was less than 10% of that in healthy individuals, and the *Clostridium* subcluster XIVab (Firmicutes) was also significantly lower [80]. Bacterial populations in IBD patients differ between different intestinal regions. For instance, in a study of 40 twin pairs with IBD, colonic CD patients had more Firmicutes than healthy subjects, while ileum CD patients had less. On a family level, colonic CD patients had an increased amount of Ruminococcaceae (Firmicutes phylum), whereas this family was reduced in ileal CD patients [81]. Nevertheless, most studies have demonstrated that Bacteroidetes bacteria exhibit pro-inflammatory properties due to endotoxins and influence cytokine production, contributing to IBD. Furthermore, Firmicutes bacteria exhibit anti-inflammatory effects and can alleviate the progression of IBD.

### 3.2. Treating Inflammatory Bowel Disease (IBD) with Probiotics

Certain probiotic strains can be used for the management of IBD by regulating gut homeostasis. Although the majority of probiotics that exert anti-inflammatory effects in IBD patients are bacteria from the phylum Firmicutes, only some of them can influence the F/B ratio. Table 2 lists *Lactobacillus* and *Bifidobacterium* genera that exhibited therapeutic properties against IBD and altered gut microbiota.

Early administration of *L. reuteri* DSM 17938 to 8-day-old C57BL/6J mice affected gut microbial homeostasis by increasing the relative abundance of Firmicutes and decreasing the relative abundance of Bacteroidetes. The probiotic gavage further increased the proportion of FoxP3+ regulatory T cells in the mice intestine while reducing their proportion in the mesenteric lymph nodes. This may indicate that *L. reuteri* DSM 17938 supports trafficking of regulatory T cells from the lymph nodes to the intestine [82]. Live and dead *L. plantarum* AN1 administered via drinking water to IBD murine models exhibited gut modulatory and anti-inflammatory properties. Both live and heat-killed bacteria increased Firmicutes and decreased Bacteroidetes abundance in the IBD murine models. The probiotic also exerted anti-inflammatory effects in vitro by producing nitrogen oxide and protecting RAW264.7 cells against hydrogen peroxide toxicity [83]. The combination of two different probiotics (*L. plantarum* ZDY2013 and *Bifidobacterium bifidum* WBIN03) reduced UC in mice through modification of gut microbiota and reduction of inflammation and oxidative stress. The probiotic mixture increased the abundance of unidentified Firmicutes and reduced the abundance of several bacterial families including Bacteroidetes (Bacteroidales S24-7). Furthermore, the two probiotics downregulated TNF-α and upregulated antioxidant factors in UC mice [84]. *L. plantarum* is one of the most commonly used probiotics against IBD. Its mechanisms of action in preventing and treating IBD are diverse [92] and include the production of bacteriocins. In IBD-induced mice models, Yin et al. compared the effects of wild-type *L. plantarum* NCIMB8826 and its mutant *L. plantarum* LM0419, which lacks the gene for the bacteriocin plantaricin. Mice receiving *L. plantarum* LM0419 had similar TNF-α and IL-6 levels as the control group receiving no bacteria, while the group receiving *L. plantarum* NCIMB8826 had lower levels of the inflammatory cytokines. Apart from reducing pro-inflammatory cytokines, *L. plantarum* NCIMB8826 (but not LM0419) also altered IBD microbial dysbiosis. The Bacteroidetes genus *Parabacteroides*, which was abundant after IBD induction, was reduced after the administration of *L. plantarum* NCIMB8826 [85]. Other lactobacilli have also proven effective in modulating dysbiosis and decreasing inflammation. *Lactobacillus fermentum* KBL374 and *L. fermentum* KBL375 altered gut microbiota in mice with dextran sodium sulfate-induced colitis. The administration of dextran sodium sulfate significantly increased the proportions of *Bacteroides* and *Mucispirillium* but decreased *Lactobacillus*. The co-administration of the *L. fermentum* KBL374 and *L. fermentum* KBL375 corrected gut dysbiosis, decreased the proportions of the two genera, and increased the proportion of *Lactobacillus*. *L. fermentum* KBL374 and KBL375 also increased the proportion of the CD4 + CD25 + Foxp3+ regulatory T cell population, decreased pro-inflammatory cytokine levels (IFN-γ, IL-4, IL-13, IL-6, TNF, IL-17A, IL-1β, CCL2, and CXCL1), and increased anti-inflammatory cytokine IL-10 levels [86].

Even though the phylum Bacteroidetes is related to inflammation and IBD, some studies indicate that certain probiotics can exert anti-inflammatory effects by increasing the abundance of Bacteroidetes. For example, the administration of *Lactobacillus casei* variety *rhamnosus* (Lcr 35) to children with acute diarrhea increased the abundance of both Firmicutes and Bacteroidetes, increasing total fecal IgA and decreasing lactoferrin and calprotectin levels [87]. Similar results were shown in another clinical trial in which IBD patients received a probiotic yogurt containing *Lactobacillus acidophilus* La-5 and *Bifidobacterium animalis* subsp. *lactis* BB-12. The mean numbers of *Lactobacillus, Bifidobacterium*, and *Bacteroides* in patients receiving yogurt were significantly increased compared to the control group [88]. In contrast, bifidobacteria-fermented milk reduced the relative proportion of *B. vulgates* from the phylum Bacteroidetes. During a one-year period of the trial, only three of 11 patients (27%) receiving bifidobacteria-fermented milk experienced exacerbation of UC symptoms, while nine out of 10 patients (90%) experienced exacerbation of symptoms in the control group [89]. Bifidobacteria also altered gut microbiota in elderly individuals. Daily consumption of 250 mL of skim-milk containing *Bifidobacterium lactis* HN019 increased the resident number of bifidobacteria, enterococci, and lactobacilli, and reduced the counts of enterobacteria [90].

The probiotic VSL#3 is among the most used probiotic products against IBD and is composed of highly concentrated live freeze-dried bacteria including four lactobacilli (*L. plantarum*, *L. casei*, *L. acidophilus*, and *L. delbrueckii* subsp. *bulgaricus*), three bifidobacteria (*B. longum*, *B. breve*, and *B. infantis*), and one *Streptococcus salivarius* subsp. *thermophilus* [93]. Its anti-inflammatory and protective effects were proven in both animal [94] and human [95] studies. However, little data on the correlation of VSL#3 with IBD and altered gut microbiota are available. Rossi et al. conducted an analysis of the anti-inflammatory effects of VSL#3 on pet dogs that were diagnosed with IBD. VSL#3 therapy significantly reduced CD3+ T cell infiltration, enhanced regulatory T cell markers (FoxP3+ and TGF-β+), and increased *Faecalibacterium* abundance [91]. As mentioned previously, *Faecalibacterium* is one of the most important Firmicutes genera in IBD, with *F. prausnitzii* exerting anti-inflammatory and protective effects in the gut [73]; its abundance is thus important in IBD patients. Apart from probiotics, the abundance of *F. prausnitzii* was increased with different prebiotics such as butyrylated high-amylose maize starch [96], inulin [97], and kiwifruit capsules [98].

Apart from contributing to balancing the F/B ratio, many probiotics reduce bowel inflammation by other mechanisms such as improving the epithelial cell barrier, modifying epithelial and immune cells, and modifying cytokine synthesis. For these studies on probiotics and their anti-inflammatory properties, the reader is referred to other reviews [99,100].

## 4. Conclusions

In this review, we summarized the properties of the two most important bacterial phyla found in the gut, Firmicutes and Bacteroidetes, and their role in maintaining homeostasis in the host. A balanced ratio between these two phyla is important for maintaining health, and alterations in the ratio are associated with the development of gut dysbiosis and certain diseases such as obesity and IBD.

Firmicutes bacteria are Gram-positive and play a key role in the nutrition and metabolism of the host through SCFA synthesis. Through their metabolic products, Firmicutes bacteria are indirectly connected with other tissues and organs and regulate hunger and satiety. In contrast, Bacteroidetes bacteria are Gram-negative and associated with immunomodulation. Their components, lipopolysaccharides and flagellin, interact with cell receptors and enhance immune reactions through cytokine synthesis.

Increased or decreased F/B ratios are associated with the development of obesity or IBD, respectively. The most frequently used probiotics in treating gut dysbiosis are bacteria from the genera *Lactobacillus* and *Bifidobacterium* as well as the yeast *S. boulardii*. Restoring the F/B ratio with the proper probiotics can reduce weight gain or suppress the immune system. Probiotics that reduce weight and decrease the F/B ratio are *L. rhamnosus*, *L. sakei*, *L. paracasei*, *L. salivarius*, *B. amyloliquefaciens,* and *S. boulardii*. Additionally, increasing the F/B ratio with probiotics is associated with immunosuppression and protective effects in the intestine. Тhe most effective probiotics with these properties are *L. reuteri*, *L. plantarum*, *L. fermentum*, *L. casei* variety *rhamnosus* (Lcr 35), *L. acidophilus*, *B. lactis*, *B. bifidum*, and VSL#3.

Overall, selected probiotics can affect F/B dysbiosis and contribute to reduced obesity and intestinal inflammation. However, different probiotics exert different effects on the F/B ratio. Choosing the appropriate probiotic strain or mixture is therefore crucial for the most optimal therapeutic effects. Further studies are needed to identify strains of Firmicutes and Bacteroidetes bacteria that play a crucial role in health and homeostasis as well as to establish approaches that modulate the concentration or composition of Firmicutes and Bacteroidetes bacteria.

## Figures and Tables

**Table 1:** Probiotics and their anti-obesity effects via F/B ratio modulation.

Probiotic	Study observations	Study Subjects and Design	Reference
*L. rhamnosus* GG, *L. sakei* NR28	Decreased F/B ratio. Reduced epididymal fat mass, acetyl-CoA carboxylase, fatty acid synthase, and stearoyl-CoA desaturase-1 in liver.	Seven-week-old C57BL/6J mice.	[39]
*L. rhamnosus* GG	Decreased F/B ratio. Prevented obesity.	Seven-week-old C57BL/6J mice.	[40]
*L. rhamnosus* hsryfm 1301	Increased Bacteroidetes and decreased Firmicutes. Reduced serum lipid levels.	Five-week-old male Sprague-Dawley rats.	[41]
*L. paracasei* HII01 + xylooligosaccharide	Decreased F/B ratio. Enhanced insulin sensitivity, decreased low-density lipoprotein cholesterol, and reduced body weight.	Male Wistar rats.	[42]
Bacteriocin-producing *L. salivarius* UCC118 *Bac+*	Increased Bacteroidetes and Proteobacteria. Decreased Actinobacteria. Non-persistent weight reduction.	Seven-week-old male C57BL/6J mice.	[43]
*L. salivarius* Ls-33	Increased Bacteroidetes-Prevotella-Porphyromonas-to-Firmicutes ratio. No reduction in body weight.	Double-blind, randomized, placebo-controlled study with obese adolescents.	[44]
*Bacillus amyloliquefaciens* SC06	Decreased F/B ratio. Reduced body weight and hepatic steatosis.	Six-week-old C57BL/6J mice.	[45]
*S. boulardii*	Decreased Firmicutes and increased Bacteroidetes. Reduced body weight, hepatic steatosis, fat mass, and inflammation.	Six-week-old leptin-resistant obese and type 2 diabetic mice.	[46]
*S. boulardii*	Decreased Firmicutes and increased Bacteroidetes. Liver protection.	Adult BALB/c mice.	[47]

**Table 2:** Probiotics and their anti-inflammatory effects via F/B ratio modulation.

Probiotic	Study observations	Study Subjects and Design	Reference
*L. reuteri* DSM 17938	Increased Firmicutes and decreased Bacteroidetes.Regulatory T cell trafficking from the lymph nodes to the intestine.	C57BL/6J mice.	[82]
*L. plantarum* AN1 (live and dead cells)	Increased Firmicutes and decreased Bacteroidetes.Anti-inflammatory effects in vivo and in vitro.	Five-week-old ICR mice.	[83]
*L. plantarum* ZDY2013 and *B. bifidum* WBIN03	Increased unidentified Firmicutes and decreased Bacteroidetes families. Increased antioxidant factors and decreased TNF-α.	Four–five-week-old female BALB/c mice.	[84]
*L. plantarum* NCIMB8826	Reduced *Parabacteroides* (Bacteroidetes genus). Reduced TNF-α and IL-6.	Six-week-old female BALB/c mice.	[85]
*L. fermentum* KBL374 and *L. fermentum* KBL375	Increased *Lactobacillus*, decreased *Bacteroides* and *Mucispirillium*. Increased anti-inflammatory and reduced pro-inflammatory cytokines.	Seven–eight-week-old female C57BL/6N mice.	[86]
*L. casei* variety *rhamnosus* (Lcr 35)	Increased Firmicutes and Bacteroidetes. Increased fecal IgA and decreased lactoferrin and calprotectin.	Randomized, case controlled study with children aged six months to six years.	[87]
Yogurt containing *L. acidophilus* La-5 and *B. lactis* BB-12	Increased *Lactobacillus*, *Bifidobacterium*, and *Bacteroides*.	Double-blind, placebo controlled study with IBD patients.	[88]
Bifidobacteria-fermented milk	Reduced *Bacteroides vulgates*. Improved UC symptoms.	Randomized controlled study with UC patients.	[89]
Skim-milk containing *B. lactis* HN019	Increased bifidobacteria, enterococci, and lactobacilli and reduced enterobacteria.	Randomized, double-blind, placebo controlled study with elderly volunteers.	[90]
VSL#3	Increased *Faecalibacterium*. Increased FoxP3+ and TGF-β+. Reduced CD3+ T-cell infiltration.	Pet dogs with IBD.	[91]
